# Experiences of out-of-hours task-shifting from GPs: a systematic review of qualitative studies

**DOI:** 10.3399/BJGPO.2021.0043

**Published:** 2021-07-21

**Authors:** Emily Lyness, Jennifer Parker, Merlin L Willcox, Hajira Dambha-Miller

**Affiliations:** 1 Primary Care Research Centre, Faculty of Medicine, University of Southampton, Aldermoor Health Centre, Aldermoor Close, Southampton, UK

**Keywords:** primary health care, after-hours care, task-shifting, workforce, out of hours

## Abstract

**Background:**

The current GP workforce is insufficient to manage rising demand in patient care within out-of-hours (OOH) primary care services. To meet this challenge, non-medical practitioners (NMPs) are employed to fulfil tasks traditionally carried out by GPs. It is important to learn from experiences of task-shifting in this setting to inform optimal delivery of care.

**Aim:**

To synthesise qualitative evidence of experiences of task-shifting in the OOH primary care setting.

**Design & setting:**

Systematic review of qualitative studies and thematic synthesis.

**Method:**

Electronic searches were conducted across CINAHL (Cumulative Index of Nursing and Allied Health Literature), PsychINFO, Cochrane, MEDLINE, Embase, and OpenGrey for qualitative studies of urgent or OOH primary care services, utilising task-shifting or role delegation. Included articles were quality appraised and key findings collated through thematic synthesis.

**Results:**

A total of 2497 studies were screened, of which six met the inclusion criteria. These included interviews with 15 advanced nurse practitioners (ANPs), three physician assistants (PAs), two paramedics, and a focus group of 22 GPs, and focus groups with 33 nurses. Key findings highlight the importance of clearly defining and communicating the scope of practice of NMPs, and of building their confidence by appropriate training, support, and mentoring.

**Conclusion:**

While NMPs may have the potential to make a substantial contribution to OOH primary care services, there has been very little research on experiences of task-shifting. Evidence to date highlights the need for further training specific to OOH services. Mentorship and support to manage the sometimes challenging cases presenting to OOH could enable more effective OOH services and better patient care.

## How this fits in

Demand for OOH GP services is increasing, but the existing GP workforce is insufficient to meet this need. Government policy proposes a greater skill mix of NMPs in the workforce to shift tasks away from GPs. Early studies suggest that NMPs deliver safe and effective care; however, there is limited evidence specific to the OOH setting. In some settings, NMPs have a defined scope of practice, which excludes certain patient groups. If this is clearly communicated, GPs will focus on more complex patients, which NMPs cannot see. This may increase GP workload within shifts but the involvement of NMPs reduces the number of shifts that GPs need to work. If the NMPs’ scope of practice is not clearly defined, communicated, or recognised, the service will run less efficiently, giving rise to negative team perceptions and poor interprofessional relationships. Building confidence of NMPs to work autonomously takes dedicated training, support, and mentoring, and is critical to efficient running of OOH services.

## Introduction

OOH primary care services deliver 'urgent care' outside normal working hours. NHS England defines urgent care as *‘* *the range of responses that healthcare services provide to people who require urgent advice and treatment’*.^1,2^ Demand for OOH services is increasing in the UK: in 2014, they received 8.6 million calls and completed 6.8 million assessments; 2.9 million via telephone, 0.9 million home visits, and 3 million face-to-face consultations, contributing to a cost of £400 million.^3^ Historically it has been challenging to establish data for comparison owing to variation in recording, changing definitions of OOH, and different methodologies of collecting this data.^4^ However, it is expected that demand will continue to rise owing to increasing levels of multimorbidity, complex health needs, and changes in service utilisation behaviour.^5–7^

The existing GP workforce is insufficient to meet OOH demand. In response, the government has proposed the development of a greater skill mix of NMPs. NMPs include advanced practitioners who are employed to undertake roles traditionally filled by GPs and can, therefore, shift tasks away from GPs. Although it is likely that task-shifting is not a novel practice, it is difficult to ascertain the current picture of task-shifting in OOH care in England owing to the wide variety in NMP roles. A 2017 study found over 500 different job titles for advance practice roles in health care and care pathway organisation.^8^

The skill mix of NMPs working in OOH has historically comprised advanced practitioners from a variety of clinical backgrounds, including primary and emergency services. The effectiveness of task-shifting from GPs to NMPs is still debated, particularly concerning managing complexity. Earlier studies suggest that NMPs are able to deliver safe and effective care with positive patient-reported outcomes, high satisfaction, and decreased costs. However, there is limited OOH specific evidence.^9–11^ One UK trial compared in-hours management of same-day requested assessments, including home visits, between GPs and ANPs and found equivalent outcomes.^10^ A Dutch quasi-experimental study examining task-shifting from GPs to nurses in OOH found similar consultation numbers, but a higher medical complexity in patients treated by GPs.^12^ Along with the acutely unwell and children aged <5 years, there is evidence that more potentially complex patient groups, such as adults aged >65 years and those with chronic diseases from socioeconomically deprived areas, are accessing care in the evenings.^6^ While this may partly be owing to barriers to daytime access, there is also a growing culture of 24-hour service demand contributing to OOH service utilisation behaviour.^5,6,13^

Furthermore, because of the variety of practitioners in OOH, there is a lack of clarity around NMP role definition and competency expectations.^14,15^ This uncertainty has led to negative team perceptions and poor interprofessional relationships with less effective service delivery.^16–19^ In turn, there are challenges recruiting NMPs to OOH settings.^19–24^

This study aimed to understand the experiences of OOH staff in addressing these issues, to find out how task-shifting can be implemented to optimise service effectiveness and patient care. Therefore, a systematic review was conducted of qualitative studies on experiences of task-shifting to NMPs from GPs in OOH primary care services.

## Method

ENTREQ guidelines^25^ were followed and the study was registered with PROSPERO.

### Search strategy and inclusion and exclusion criteria

A pre-planned systematic literature search was performed (database inception to October 2020) across CINAHL, PsychINFO, Cochrane, MEDLINE, Embase, and OpenGrey. No restrictions on publication date, country, or language were used. In addition to the electronic database search, references of included studies were manually searched for relevant articles. The detailed search strategy is provided in Supplementary Table S1. Experts in eight countries (Austria, Denmark, Germany, the Netherlands, Norway, Sweden, UK, and US) were also contacted in April 2021 to ask whether they knew of any relevant studies. Inclusion criteria were empirical studies with qualitative methods or mixed methods with a qualitative component reporting on task-shifting from GPs to NMPs (including nurses, paramedics, and PAs) within the OOH primary care setting (outside of routine opening hours).

### Study selection and data extraction

All titles and abstracts were initially screened by a single author, then two authors independently screened a 10% sample.^26^ Full-text versions of potentially eligible articles were reviewed in duplicate and any ambiguity resolved by team discussion (see Supplementary Figure S1). Data were extracted from the results sections of included articles using NVivo (version 1.3) in preparation for thematic synthesis.

### Results synthesis

Thematic synthesis was undertaken as outlined by Thomas and Harden,^27^ including coding of text line by line followed by the development of descriptive themes, and then generation of analytical themes. Each article was initially read and relevant qualitative data highlighted to produce a conceptual framework. This allowed iterative formation of codes relating to experiences of OOH task-shifting. Line-by-line examination of the data allowed inductive exploration and identification of recurring patterns describing individual experiences and perspectives. Common themes were identified in team discussion, which built on the framework. Findings were then summarised using a narrative approach and were drafted into a summary by one author, then commented on by the other authors. Through several rounds of discussions, an iterative process of hierarchical ordering of themes was repeated until analytical themes describing and/or explaining initial descriptive themes were present, and team consensus reached. This ordering of themes enabled a deeper understanding of the unifying concepts to explain experiences of task-shifting in OOH.

### Risk of bias

The Critical Appraisal Skills Programme (CASP) assessment tool for qualitative research^28,29^ was used by two researchers to independently appraise the studies; disagreements were resolved by discussion. This assessment was not used to judge weight of findings, or exclude studies, but to highlight potential bias in the results of included studies.

## Results

### Study selection

The search identified 2497 articles (see Supplementary Figure S1). After removal of duplicates and screening, 42 articles were selected for full-text screening. Team discussion excluded a further 36 articles because they were not about OOH activities such as home visits or consultations. Six articles were included in the review. Study characteristics are summarised in Supplementary Table S2. Four were small UK-based, mixed-methods studies with qualitative components, published between 2011 and 2019; two reported experiences of ANPs conducting home visits in Bristol and Cumbria;^11,19^ one reported a pilot of integrating US-trained PAs into an OOH service in Scotland;^30^ and one reported the evaluation of a programme to train paramedics to work in OOH in Bristol.^31^ One study in Norway investigated the experiences of nurses triaging patients presenting with respiratory symptoms,^32^ and one study in the Netherlands explored the views of GPs and nurse practitioners (NPs) taking part in a quasi-experimental study into optimal skill mix in OOH primary care.^33^

### Quality of included studies

The CASP quality assessment (see Supplementary Table S3) identified weaknesses in the reporting of recruitment strategy by three studies.^19,30,31^ Farmer *et al* did not specify how many PAs were included in focus groups nor how they were recruited, but an appropriately broad sample was described.^30^ Farmer *et al* also omitted reporting of the interview method, data management, and analysis approach, which limits appraisal of whether this data addressed the research question.^30^ Moule *et al* provided a limited description of the thematic approach used to analyse the responses of the three interviews carried out.^31^ Yuill did not state the qualitative analysis technique employed.^19^ The reflexivity of the research team was not reported by four studies.^19,30,31,33^ Ethical issues were not fully explored by Lindberg *et al*.^32^

### Summary of themes

Two main factors were identified that influenced both job satisfaction and efficiency of NMPs in OOH services: 1) a clearly defined skillset, scope of practice, and role; and 2) confidence (see Supplementary figure S2). These could be enhanced by clear communication of the roles, recognition and collaborative working by GPs, and by training, support, and mentorship from GPs. Additional illustrative quotes are available in Supplementary Table S4.

#### Theme 1: Clearly defined skillset, scope of practice, and role of NMPs

##### Defining scope of practice and role

Some OOH services appeared to expect NMPs to function as GPs, although they do not have the same breadth and depth of knowledge and skills. Therefore, it is not surprising that they felt unprepared for certain patients and sometimes avoided them if possible:

*‘We tend to see them all as equal, but they do have different experiences and abilities.’* (GPs talking about ANP home visiting)^11^

*‘*[ANPs] *mentioned that certain groups of patients are more complex than others and their experience had not always prepared them for this. Common areas included palliative, children’s and mental health care, providing telephone advice, managing comorbidities and wider prescribing* … [they] *would avoid specific patient groups, including children and people with mental health issues, but they would see such patients if they were the only clinician on the shift.* *’* ^19^

Rather than expecting NMPs to see all patients, the GP cooperative studied in the Netherlands specifically defined certain patient groups as outside the scope of practice of NMPs: patients aged <1 year, patients suffering psychiatric complaints, abdominal pain, chest pain, a neck ailment, headache, or dizziness.^33^ This enabled the service to run efficiently as long as GPs accepted and collaborated by focusing their work on patients that NMPs could not see:

*‘If there are twoNPs we have to check all patients on the presentation list, not only their urgency level, but also their complaint. If it’s, for example, an ear infection, I take the next patient and leave the ear infection to theNP.‘* (GP in OOH cooperative)^33^

Van der Biezen *et al* described a clear trade-off in terms of the impact on GP workload of employing more ANPs. When ANPs worked more shifts, GPs’ workload was reduced in terms of the number of shifts they were required to work; however, during shifts with more ANPs, GPs’ workload was increased in terms of longer consultations with more complex cases.^33^ On the other hand, NMPs brought some unique skills, which in some cases enabled them to work more efficiently than GPs:

*‘NPs ask less often for support from the medical assistant compared to GPs; for example, regarding putting on bandages after suturing* *.’* ^33^

##### Recognition of NMPs’ skillset and role

It is important that colleagues recognise the unique skills offered by NMPs and their role. Greater appreciation of different NMP training backgrounds could avoid potential misunderstandings that occur when roles are viewed as equivalent. In some cases, GPs did not understand the scope of practice of ANPs. ANPs described needing to gain the trust of colleagues in primary and secondary care, who were uncertain about the NMPs’ roles. They had some negative experiences of patient contact, especially if clinical triage had first been carried out by a GP:

*‘A lack of understanding about the ANP title and competencies, by patients and other members of the public as well as colleagues including GPs, nurses and other primary and secondary care staff* … *this is exacerbated by the wide variety of people calling themselves advanced practitioners or nurse practitioners who do not necessarily have the requisite experience or qualifications to undertake the role.* *’* ^19^

Greater clarity of role expectation enabled NMPs to feel valued and satisfied in their jobs, and clearer communication was needed to ensure the whole team was informed:

*‘* *PAs reported working most effectively and were most satisfied where there was a distinct gap in a team that they could fill; for example, in the OOH clinic they worked like a supporting GP to a lead GP.* *’* ^30^

*‘At the start of the shift, I say,“I don’t treat those patients“ … then you start doing consultations. Making agreements with the GP who starts later isn’t really working, you’re either busy yourself, or the door of the GP is already closed.‘* (NP in OOH cooperative)^33^

Understanding the scope of practice and limitations of NMPs was emphasised as critical to avoid inappropriate task allocation and to ensure the service runs efficiently:

*‘Some shifts run perfectly, others you think“I wish there was a third GP now.”Especially when there are GPs who pick out patients with skin complaints from the presentation list. You get delay if more patients with abdominal pain show up.‘* (Medical assistant in OOH cooperative)^33^

#### Theme 2: NMP confidence

##### NMP confidence is critical for an efficient service

Lack of confidence was commonly reported by NMPs, leading to anxiety and inefficiencies in the service. Nurses conducting telephone triage reported offering GP appointments to some patients who didn’t need them, because they were worried about missing serious illness, and lacked the confidence to reassure patients and their parents over the phone.^32^ This led to both slower triage (as the nurses felt the need to document the history in detail) and excessive numbers of GP appointments:

*‘You never sit in that chair and feel confident.’* (ANP doing telephone triage)^32^

Lack of confidence was also given as a reason for NMPs working more slowly in face-to-face consultations:

*‘I must say a patient with, for example, a sore, I finish those consultations within a minute and I feel confident to do that. I think NPs are more careful and still take a full history just to be sure they do their work properly.‘* (GP in OOH cooperative)^33^

Some nurses also described lacking confidence in making referrals to secondary care:

*‘For nurses, having the confidence to talk to doctors about complex presentations and diagnoses can be very challenging, but once you get used to it, it becomes easier* *… ‘* ^19^

Inadequate preparation could exacerbate challenges faced by NMPs and contribute to a feeling of isolation when starting roles in OOH teams:

*‘Daytime* [practice has] *more protection and barriers* *…* *the step into OOH was a big one* *… unknown* *… no exposure to what is being seen* [with] *more acute unwell patients* *.* *’* (ANP home visiting)^11^

##### Training, clinical support, and mentorship help to build confidence and autonomy

Individualised training and support were suggested as key for NMPs who may be less experienced in traditional GP tasks, particularly at the beginning of a career. However, it was recognised that the need decreased with time as NMPs gained experience. Investing in role-specific training, particularly at induction, could minimise potential role uncertainty and help NMPs feel valued as part of the professional team:

*‘Working autonomously in home visits, ANPs reported:”when you start in an organisation, you don’t know who to go to* ... *as you learn, you know who you can go to for support* *.* *”* *There was consensus among the interviewees that having some protected time for learning on- and off-shift would be beneficial* … *”* *makes them feel valued and that we do care about their continuing professional development.* *”* *’* ^19^

NMPs viewed the clinical training required to undertake their roles as an opportunity for career development, while maintaining clinical practice. Practitioners who are trained to specified professional standards are able to take more clinical responsibilities, which facilitates task-shifting:

*‘* *It was felt that providing training on a sliding scale would be useful for different healthcare practitioners. A nurse experienced in independent practice in another environment may only need* *8* *-* *weeks* *training to achieve the competencies; however, paramedics may need* *16* *weeks* *to achieve the competencies.* *’* ^31^

Professional dialogue, case discussion, and peer learning within the OOH team could minimise professional hierarchy, especially within nurse–doctor relationships:

*’Peer support was mentioned as positive* … *participants talked about learning from clinical colleagues, sharing experiences* … *one* [ANP] *mentioned that* *:* *“* *Nurses always think hierarchically, that GPs are better than you is the thought/perception, but when you are learning together* … *you realise that they don’t know things, so you are like them, which breaks down the barriers.”’* ^19^

Clinical mentorship was flagged as an effective way to support NMPs in a different way to clinical training. This should be regular, consistent, and accessible, particularly in the first year of practice, but also throughout their professional career development:

*‘ANPs acknowledged that the knowledge and support of the GPs was important, although with time this was only required occasionally.’* (ANP home visiting)^11^

*‘GPs and ANPs suggested:“a structured ANP home-visiting induction…needs to be developed to include typical home-visit scenarios, shadow shifts, and a mentorship programme* … *the opportunity for clinical supervision, case-review discussion, ongoing audit of cases, and a forum to debrief* *.”’* ^11^

Specific support from a designated GP was considered beneficial in ensuring NMP confidence. This may promote job satisfaction and reduce anxiety for those who lack confidence even to approach a GP to ask for support:

*‘*[Paramedic] *students recommended concentrated time spent with one allocated mentor and that GP support is increased.’*^31^

NMPs wanted to practice autonomously and found this to be an attractive aspect of their role; however, it could also provoke stress. Therefore, having access to GP support was still necessary to provide reassurance, validation of clinical decisions, and to build confidence and autonomous practice:

*‘*[Anxiety] *about working alone, knowing that support, often by phone, was available but not always immediately contributed to a feeling of isolation and stress as they experienced the need for autonomous decision making...‘*^19^

*‘Some* [ANPs] *said one of the most enjoyable aspects was being able to make decisions* *“knowing your* *"* *own sphere of competence* *"* *, treating patients within this and referring on as appropriate were important* *.”’* ^19^

## Discussion

### Summary

There have been very few qualitative studies on experiences of task-shifting from GPs to NMPs in the OOH primary care setting. This study highlights the importance of clearly defining NMPs’ roles and scope of practice. In addition, the importance of providing training, support, and mentoring is shown in order to develop their confidence to practice autonomously and efficiently.

### Strengths and limitations

A comprehensive electronic search of the literature was conducted, complemented by grey literature, manual searching, and contacting experts. Although 2497 papers were screened, only six matched the inclusion criteria, from only three European countries. The system of OOH primary care varies in different countries so it is possible that task-shifting activities to NMPs have long existed in many countries and is, therefore, not novel, resulting in minimal clinical research published in this area.^34^

The low numbers of participants included in the studies raises the possibility of recruitment bias and limits transferability of these findings, even within the UK. Additionally, not all qualitative studies included were of high quality, particularly those that were part of a mixed-methods approach. However, this reflects the available literature and emphasises the need for more research around NMPs and OOH task-shifting.

The review team included GPs with experience of OOH work. While this provides strength to the analytical approach and contextual understanding of the subject, it may have affected interpretation of findings through the authors' assumptions made based on their own experiences; however, the authors attempted to guard against this by strictly limiting themselves to what was reported in the included studies.

### Comparison with existing literature

NMPs can deliver safe and effective in-hours primary care, positive patient-reported outcomes, and decreased organisational costs, but there is limited data on long-term outcomes for medical complexity and multimorbidity.^14,22–24,35^ The findings of the present study suggest appropriate training, mentorship, and interprofessional collaboration can enable NMPs to feel supported in the management of a wider variety of cases in OOH, which promotes job satisfaction, professional development, and efficiency of the service. These findings are consistent with the recognition that NMPs, specifically nurses, desire collaboration to mitigate potential imbalances in workload and dissatisfaction that can occur when working in a subsidiary role. The concept of complexity itself is a continuum dependent on the clinical presentation, and there is evidence that ANPs may be more likely to manage those socially complex cases,^36^ while GPs focus on medically complex patients. Thus, a collaborative approach is likely to be useful and relevant to the OOH population.

Much of the evidence for NMP task-shifting comes from non-urgent care such as nurse-led chronic care.^20,35,37–39^ The Royal College of General Practitioners and Health Education England have designed a Core Capabilities Framework for advanced practice in primary care.^40^ This brings into alignment nursing professionals in primary care. Although not specific to OOH, there is overlap. This framework could reduce uncertainty about NMP role definition and scope of practice.

However, for those NMPs already in the OOH workforce, there remains some confusion about professional identity and respect for clinical autonomy. Professional identity not only encompasses clinical autonomy, but also confidence and aspiration in professional development. The literature examining role transfer to advanced practitioners supports the importance of mentorship and supervision. This is likely to be vital in encouraging retention of NMPs and encouraging professional development into leadership roles, thus contributing to the sustainability of the OOH workforce.^20,23,39,41^

### Implications for research and practice

NMPs have the potential to support growing OOH workloads through task-shifting. Additional support must include context-specific role preparation, clinical mentoring, and a collaborative approach to sharing the caseload efficiently. Standardised core competency frameworks incorporated into current training would engender uniformity and trust in the advanced practitioner role in OOH primary care, and would be relevant to other healthcare settings where NMPs share tasks. However, more research is needed to better understand the optimal team composition, taking into account the trade-off between decreasing the number of GP shifts and increasing the workload for GPs during shifts when more NMPs are working. Where scope of practice has been defined by some OOH services, this is not consistent and appears somewhat arbitrary. Better evidence is needed to underpin decisions and definitions regarding scope of practice for NMPs in OOH services. Further qualitative research is needed into the experiences of NMPs (especially paramedics and PAs) in a wider range of OOH services in different countries. It is important to improve understanding of the barriers and enablers to service efficiency and NMP job satisfaction in a range of roles, including not only home visits, but also face-to-face and telephone triage consultations.

While NMPs have the potential to make a substantial contribution to the OOH setting, understanding the experiences of those involved in task-shifting and addressing their specific professional training needs is vital to prepare NMPs for the OOH setting. Clear definition and communication of roles, coupled with mentorship and support, could improve efficiency of OOH services and patient care. More evidence is needed to fully understand the experiences of OOH staff undertaking task-shifting in OOH primary care and how this can be improved.

## Trial registration number

PROSPERO (reference number: CRD42020218866)
